# Twist in the Tail: Escape from HIV Neutralising Antibodies at a Single Site Confers Broad Susceptibility to Others

**DOI:** 10.1016/j.ebiom.2016.09.019

**Published:** 2016-09-20

**Authors:** Adam K Wheatley, Stephen J Kent

**Affiliations:** a Department of Microbiology and Immunology, University of Melbourne, Peter Doherty Institute for Infection and Immunity, Melbourne, Victoria, Australia; b ARC Centre of Excellence in Convergent Bio-Nano Science and Technology, University of Melbourne, Melbourne, Australia; c Melbourne Sexual Health Centre, Department of Infectious Diseases, Alfred Health, Central Clinical School, Monash University, Melbourne, Australia

An efficacious vaccine for HIV remains elusive. Numerous groups have isolated antibodies from HIV infected individuals that can bind and neutralise antigenically diverse HIV strains, so-called broadly neutralising antibodies (bNAbs) (Wibmer et al., 2015). HIV bNAbs target several conserved regions on the viral envelope (Env; a heterotrimer composed of gp120 and gp41 subunits) including the CD4 binding site, peptidoglycan associated with either the V1/V2 or the V3 loops, and the gp120-gp41 interface or the membrane proximal external region (MPER) of gp41. Possessing broad and potent antiviral activity, bNAbs may have utility in HIV immunotherapy, and are also an attractive template to guide the rational design of vaccines to generate analogous humoral immunity. However, bNAbs arise in only a minority of infected individuals and have been associated with extended periods of viremic infection and more recently, perturbations in the follicular and regulatory CD4 T cell compartment (Moody et al., 2016). Analysis of isolated bNAb lineages reveals genetic and structural features that likely contribute to their scarcity, including very high rates of somatic mutation, restricted germline selection, frequent genetic insertions and deletions, extended CDR-H3 regions and a propensity for poly- or autoreactivity. The complicated immunological contexts that underpin bNAb development are unlikely to be recapitulated by vaccination. Indeed, generating serum antibody responses able to combat neutralisation-resistant viral isolates (so-called tier 2 viruses) has not been consistently demonstrated by immunisation. Clearer insights into what governs neutralisation sensitivity to bNAbs should help speed further development of bNAb-based immunisation strategies and HIV immunotherapy.

In the current issue of *EBioMedicine*, Bradley et al. (2016) characterise changes in the gp41 MPER that render HIV isolates with neutralisation-resistant phenotypes sensitive to a range of bNAbs. In a pair of South African individuals infected with clade C virus who developed broadly neutralising antibodies, they identified viral isolates with amino acid changes in the MPER at W680 and Y681 that bestowed resistance to MPER-targeting neutralising antibodies. Interestingly, substitutions at these positions conferred increased sensitivity to bNAbs binding the CD4 binding site or V3 loop regions of Env, some distance from the MPER region. Anti-MPER antibodies isolated from these individuals failed to bind the mutated HIV isolates, suggesting that these viral variants arose in the face of immune pressure from the early autologous neutralising responses. A number of previous studies established that MPER modifications modulate neutralisation sensitivity at distal sites ((Back et al., 1993) and others). However, Bradley et al. comprehensively demonstrate increased susceptibility to heterologous clade C sera, CD4-mimetics, bNAbs and interestingly, vaccine-elicited antibodies in macaques. Hence residue substitutions at positions 680 and 681 within MPER appear to elicit conformational shifts in the Env trimer to favour open structures amenable to neutralisation at other sites, including exposure of both the CD4 binding site and the CCR5 co-receptor binding site in V3. The detailed delineation of specific conformational changes await confirmation by structural biology and/or crystallography.

The complex interplay between viral escape and Env conformation observed by Bradley et al. and others gives some pause to reductionist approaches focussing on recapitulating single bNAb specificities by immunisation. Favourable linkage interactions between different bNAb epitopes, shown here for the MPER and the CD4 binding site or V3 loop, suggests vaccines simultaneously targeted to multiple epitopes may be advantageous. While targeting the MPER by vaccination may be difficult due to described self-mimicry and frequent generation of autoantibodies (Haynes et al., 2005, Williams et al., 2015), the results suggest effective MPER responses may complement neutralisation at alternative epitopes by constraining potential pathways of viral escape. This is supported by observations that combinations of two or more HIV bNAbs may drive some synergistic neutralisation activity beyond the simply additive (Kong et al., 2015). Similarly, rapid emergence of neutralisation resistance following bNAb monotherapy in HIV infected individuals was recently reported (Lynch et al., 2015, Caskey et al., 2015), further highlighting how synergistic bNAb combinations will be required for HIV therapy. From the perspective of vaccine immunogen design, the study by Bradley et al. highlights the importance of gp41 MPER residues near the viral membrane in maintaining the stability of the closed native trimer state. However, it is notable that the exceptionally well-characterised stabilised Env trimer BG505 SOSIP (truncated at residue 664) lacks this MPER region and has been experimentally confirmed to exist in a closed, neutralisation resistant state (Sanders et al., 2013). Future studies aimed at accurately defining the complex determinants of neutralisation sensitivity will be informative for both preventive HIV vaccine immunogen design and for the application of combination HIV bNAb therapy in HIV infected subjects.

## Conflicts of Interest

The authors declare no conflicts of interest.
